# Hydrometrocolpos and postaxial polydactyly in a girl newborn: A case report

**DOI:** 10.1002/ccr3.5453

**Published:** 2022-02-16

**Authors:** Morgan L. Day, Crystal C. Avila, Dawn L. Novak

**Affiliations:** ^1^ Department of Pediatrics University of New Mexico Children's Hospital Albuquerque New Mexico USA; ^2^ Department of Neonatology University of New Mexico Children's Hospital Albuquerque New Mexico USA

**Keywords:** Bardet–Biedl Syndrome, hydrometrocolpos, McKusick–Kaufman Syndrome, post‐axial polydactyly

## Abstract

This case report is of a 35‐week female neonate with a cystic abdominal mass. Physical examination was notable for post‐axial polydactyly, distended abdomen, and abnormal urethral opening. Differential diagnosis includes Bardet–Biedl Syndrome (BBS), an autosomal recessive ciliopathy. Genetic panel revealed she was a carrier for a BBS mutation.

## Graphical abstract

A newborn presenting with hydrometrocolpos and postaxial polydactyly should be considered for Bardet–Biedl Syndrome (BBS). These infants require close, routine follow‐up due to the potential for severe renal and retinal disease early in life.

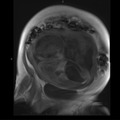

## 1 CASE PRESENTATION

**FIGURE 1:**
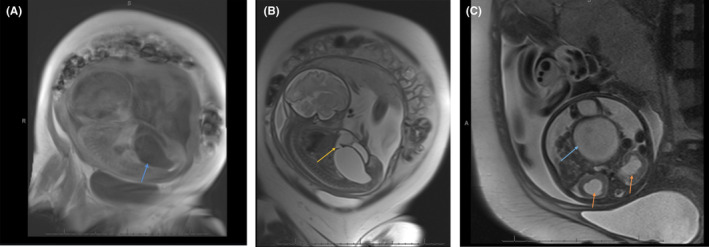
(A) Abdominal cyst appreciated in maternal/fetal MRI (arrow). This finding prompted further imaging with fetal MRI. (B) Abdominal cyst with fistula connecting to another cystic structure (arrow). The presence of a cystic abdominal mass raised concern for Bardet–Biedl Syndrome during prenatal care, and prompted the family to deliver at our institution where NICU and pediatric urology services were available. (C) Abdominal cystic mass (blue arrow) with bilateral hydronephrosis (red arrows). In addition to the abdominal cystic mass, hydronephrosis secondary to urinary tract compression is commonly seen in patients with Bardet–Biedl Syndrome

A girl infant was born at 35 weeks gestation in breech presentation via cesarean section to a 31‐year‐old G2 P2 woman. Pregnancy was complicated by gestational diabetes mellitus 2, gestational thrombocytopenia, and polyhydramnios. Antenatal ultrasound at 28 6/7 weeks gestation revealed a fetal abdominal cystic mass, which had been monitored closely and followed by fetal/maternal magnetic resonance imaging (MRI). Imaging revealed findings consistent with hemato/hydrometrocolpos and mild volume ascites likely secondary to bilateral fetal hydronephrosis Figure 1A‐C. Prenatal screening laboratories were unremarkable. Infant's birth weight was 2910 g with initial Apgar scores of 3 and 9 at 1 and 5 minutes, respectively. The infant was noted to have a significantly distended abdominal circumference and experiencing respiratory distress ultimately requiring endotracheal intubation.

**FIGURE 2:**
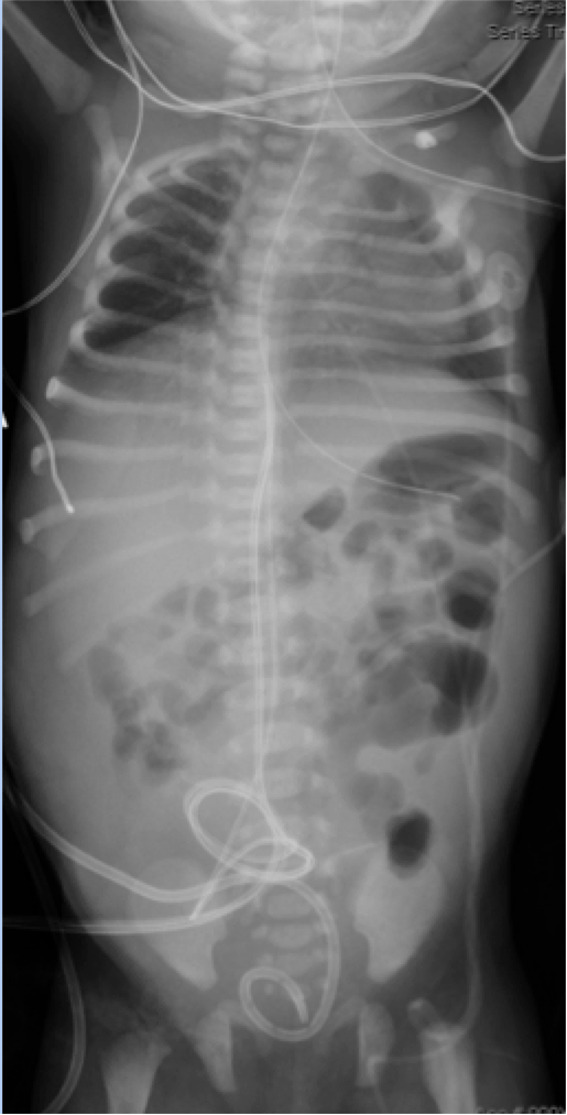
Hypoinflated lungs. This is another common finding of infants born with concern for Bardet–Biedl syndrome as the protruding abdomen impact lung development in utero

**FIGURE 3:**
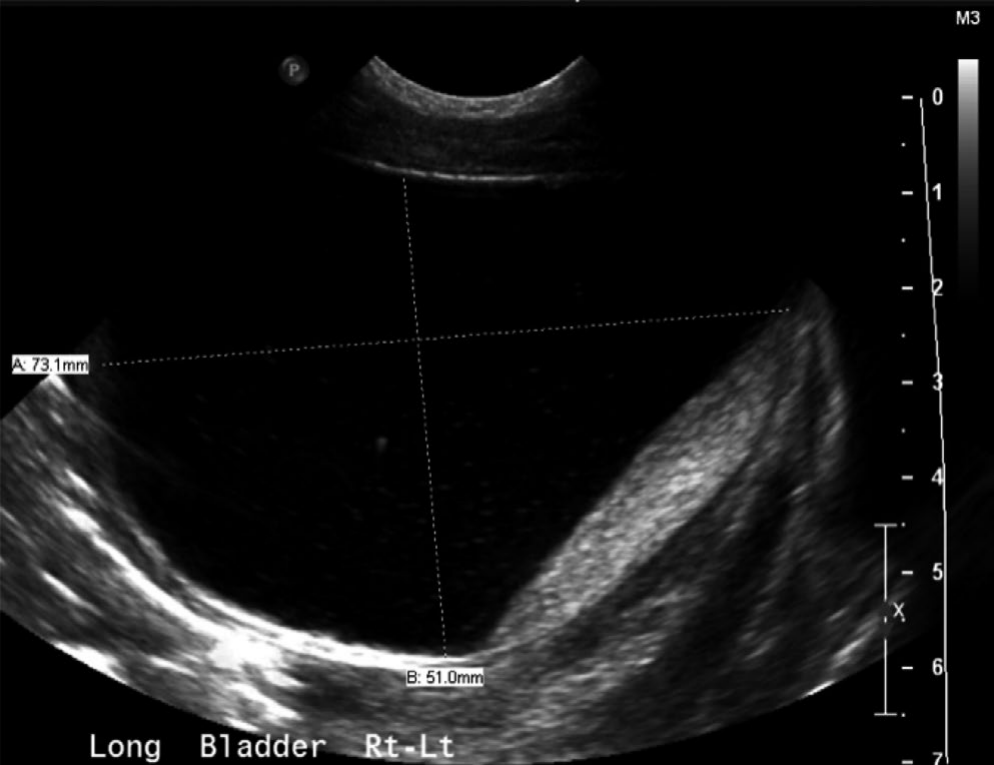
Measured bladder. Distended bladder is commonly seen secondary to outflow obstruction from urogenital sinus abnormalities

**FIGURE 4:**
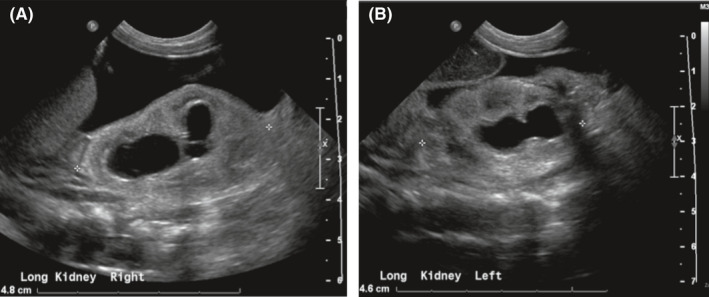
(A) Right hydronephrosis demonstrated on renal ultrasound. (B) Left hydronephrosis demonstrated on renal ultrasound

Chest radiography following intubation showed hypoinflated lungs Figure 2. Abdominal ultrasound was completed at 4 hours of age and revealed moderate to severe bilateral hydronephrosis thought to be secondary to a pelvic mass as well as moderate pelvic ascites Figure 3; Figure 4A,B. The infant had no urinary output in the first 6 hours of life. On physical examination, she had a palpable abdominal mass and significant abdominal distension. Bedside nursing attempts to place a foley catheter were unsuccessful. Thereafter, urology was consulted for catheterization; however, after several unsuccessful attempts with varying catheter sizes, interventional radiology was consulted who ultimately placed a suprapubic catheter, removing a total of 90 cc of fluid. Creatinine was measured confirming the fluid to be urine.

Physical findings were remarkable for left hand and right foot post‐axial polydactyly, otherwise, the infant appeared non‐dysmorphic with normal gross appearing girl genitalia. She was in the 82nd percentile for weight, 35th percentile for length, and 92nd percentile for head circumference, based on the Fenton girls growth chart. Her abdominal circumference at birth was 36 cm. Newborn screening results were significant for elevated TSH; however, this was believed to be transient. A genetic panel was obtained to assess for a genetic mutation that can be associated with hydrometrocolpos.

On day of life (DOL) 2, genetics was consulted and recommended DNA testing with a multi‐gene BBS panel, which also includes the MKKS gene. Genetic testing revealed that this infant is a carrier of the BBS12 associated disorder, but cannot confirm the diagnosis of BBS at a molecular level. Therefore, she requires follow‐up by the geneticist to monitor for the potential development of syndromic features associated with BBS. Patient is going to require close outpatient follow‐up by ophthalmologists to monitor for development of retinitis pigmentosa.

As previously mentioned, the infant required a percutaneous drain to be placed due to failed urinary catheter attempts and inability to spontaneously void. After urinary drainage, infant's respiratory status improved and was weaned to room air on DOL 3. On DOL 4, the infant had a cystoscopy and a vaginostomy was created. This procedure decreased her abdominal circumference by 7–29 cm. On DOL 8, the infant had increasing abdominal distension and urinary obstruction and was taken to the operating room to close the vaginostomy and create a vesicostomy. Thereafter, the infant had a stable abdominal circumference of 30–31 cm. The infant continues to require hydrocolpos irrigation and drainage once a month and is followed by urology with future plans of an anatomical corrective surgical procedure.

## 2 DISCUSSION

The differential diagnosis for this girl infant with the finding of hydrometrocolpos on fetal MRI is Bardet–Biedl Syndrome (BBS) versus McKusick–Kaufman Syndrome (MKKS). ^1^, ^2^ Additionally, the findings of post‐axial polydactyly (PAP) in the setting of genitourinary malformations further increased clinical suspicion of these two syndromes.^1^, ^2^ Hydrometrocolpos is a rare finding and develops in the setting of a congenital vaginal obstruction (i.e., vaginal atresia, transverse membrane of the vagina). ^1^ This causes accumulation of secretions in the fetal abdomen, which presents as a cystic pelvic mass, as seen in our patient.^3^ Ureterohydronephrosis secondary to urinary tract compression is commonly seen.^1^, ^3^, ^4^ Hydrometrocolpos may be the sole finding, or may present with other genital malformations, such as urogenital sinus, or with extra‐genital malformations.^2^, ^5^, ^6^, ^7^ Male infants with polydactyly may present with hypogonadism and other urinary tract anomalies.^2^ Our patient's abnormal ureteral opening, as evidenced by multiple failed foley catheterization attempts, is consistent with other case reports of BBS/MKKS, which have described ureteral openings in the vagina.^8^ The most common non‐genital anomaly associated with the finding of hydrometrocolpos is post‐axial polydactyly (PAP), occurring in up to 95% of patients with BBS.^1^, ^2^, ^5^, ^9^ Less commonly, cardiac and gastrointestinal malformations may also be seen.^1^, ^2^, ^5^, ^6^, ^7^

Bardet–Biedl Syndrome (BBS) is a rare autosomal recessive ciliopathy, which manifests as retinal degeneration/dystrophy, genital malformations, polydactyly, obesity, renal dysfunction, and cognitive impairments.^2^, ^5^ Prevalence is about 1:100,000 in North America and Europe.^10^ Diagnosis of BBS can initially overlap with MKKS, where hydrometrocolpos and polydactyly are also present.^2^, ^5^, ^6^ Definitive diagnosis is required, as the common BBS manifestations of retinal disease, learning disability, obesity, and renal failure have not been documented in a patient with MKKS.^1^, ^2^, ^3^ The majority of patients meet clinical criteria for BBS by the age of 9 years old, with some features (primarily obesity) developing around 2–3 years of age.^1^, ^5^, ^6^, ^11^ BBS can be diagnosed with either four primary criteria (rod‐cone dystrophy, limb defects, obesity, learning difficulties, and renal tract anomalies) or three primary criteria plus two secondary criteria (speech disorder, cataracts, astigmatism, ataxia, diabetes mellitus, dental crowding, congenital heart disease, or hepatic fibrosis). ^5^

There are currently 21 genes (BBS1‐ BBS21) mapped to various chromosomes that are responsible for BBS.^9^, ^10^ Of these genes, *BBS1* and *BBS10* are the most mutated, and account for 51% of cases in Northern Europe, and 20% of cases in North America, respectively.^10^ Genetic analysis alone cannot establish a definitive diagnosis of BBS, as there is overlap between the mutations found in BBS and MKKS.^6^, ^10^ Additionally, phenotypic variability makes it difficult to predict which diagnostic features will manifest even if the specific genetic mutation is known.^4^, ^10^, ^12^ Genotyping can be helpful with regards to recurrence risk, which can be as high as 25%.^12^ This heterogeneity makes follow‐up for infants presenting with hydrometrocolpos and polydactyly imperative..^10^

Renal anomalies are a major cause of morbidity and mortality in patients with BBS.^4^, ^10^, ^13^ Historically BBS has been associated with polycystic kidney disease, which is typical of ciliopathies with renal manifestations.^10^ Other structural defects affecting patients with BBS include atrophic/scarring, echogenic or loss of corticomedullary differentiation, cystic or dysplastic kidneys, other developmental abnormalities, or hydronephrosis.^4^, ^14^ New literature suggests that renal function is highly variable^4^ and dependent on maintenance of secondary causes of renal disease, specifically metabolic syndrome, hypertension, and diabetes.^10^ The prevalence of renal disease is estimated to be between 50% and 80%, with 8% of these patients requiring dialysis for end‐stage renal disease or transplantation.^10^ The majority of patients who develop ESRD do so before the age of 5 years.^10^ Unfortunately, retinal degeneration is the clinical manifestation of BBS with the strongest penetrance, affecting up to 90% of patients.^13^ Infants with BBS develop retinitis pigmentosa within the first decade of life, and become legally blind by the second to third decade of life.^2^, ^13^ Night blindness is the most common presenting visual symptom, usually manifesting around 8.5 years of age.^10^

Current recommendations for care of infants with BBS are based on known complications and manifestations of disease. These include baseline electroretinogram (ERG) and visual evoked potentials (VEP), renal ultrasounds, intravenous pyelogram (IVP), echocardiography, speech assessment and therapy; with semi‐annual urine analysis and annual blood pressure, serum urea and creatinine, blood sugar, lipid profile, and liver function tests.^12^ Management of BBS currently focuses on aggressive maintenance of diabetes, hypertension, and metabolic syndromes, with the goals of preserving function in eyes and kidneys.^10^

## 3 CONCLUSION

Antenatal hydrometrocolpos is a rare radiologic finding. Presence of hydrometrocolpos should raise suspicion for BBS or MKKS, and mandates birth of infant at appropriate facility with NICU capabilities and pediatric urologic surgery service. Genitourinary anomalies and limb defects are the most common associated symptoms of BBS and MKS. Because of the genetic overlap between BBS and MKKS, diagnosis of either syndrome is a clinical one based on presence or absence of diagnostic criteria, and is usually made around 2–3 years of age. Definitive diagnosis cannot be made on genotyping alone. Routine screening for renal function and eye examinations is imperative as end‐stage renal disease can develop before the age of 5 years, and night blindness can occur within the first decade of life. Due to multisystem nature of Bardet–Biedl syndrome, routine follow‐up is paramount and parents/caregivers need to be educated on manifestations of this disease.

## CONFLICT OF INTEREST

The authors have no conflict of interest to disclose.

## AUTHOR CONTRIBUTIONS

All authors were involved in the treatment of the patient and counseling of her family members. The first author MLD (Morgan L. Day) performed the literature review, wrote the case discussion and conclusion, and participated in editing the manuscript. The second author CCA (Crystal C. Avila) collected all the required case information, including the images, and wrote and edited the patient presentation. The third author DLA (Dawn L Novak) reviewed and edited the final manuscript, and provided mentorship and guidance throughout the project.

## CONSENT

The authors obtained written consent from patient's legal guardians to publish this case report prior to submission.

## Data Availability

Data sharing is not applicable to this article as no new data were created or analyzed in this study.
